# Diabetic emergencies presenting during the COVID‐19 pandemic: A retrospective case analysis

**DOI:** 10.1002/edm2.313

**Published:** 2021-10-29

**Authors:** Tobin Joseph, Eleanor Crawley, Sulmaaz Qamar, Maya Zosmer, Girish Rayanagoudar, Chidambaram Nethaji, Ravi Menon

**Affiliations:** ^1^ Hammersmith Hospital London UK; ^2^ Royal Free Hospital London UK; ^3^ North Middlesex University Hospital London UK

**Keywords:** COVID‐19, diabetes mellitus, diabetic emergencies

## Abstract

**Introduction:**

COVID‐19 has triggered a global pandemic and is an emerging situation. Diabetes has been associated with significant mortality in SARS and MERS‐COV infections. Patients with diabetes are at risk of COVID‐19 triggering diabetic emergencies due to known and unknown mechanisms. There is little evidence overviewing the clinical course of COVID‐19 patients who either present or have diabetic emergencies during their disease course.

**Methods:**

We conducted a retrospective case analysis of all patients admitted to our hospital during the COVID‐19 pandemic. The inclusion criteria were all patients receiving treatment for COVID‐19 and either presenting with a diabetic emergency on admission or developing an emergency during their admission. Data collected for the study were all routinely collected data as part of the admission. We compared these data to nine patients with no COVID‐19.

**Results:**

Thirty patients received treatment for a diabetic emergency, of which 21 also received treatment for COVID‐19. Significant differences were found between pH and bicarbonate on admission between RT‐PCR‐positive and both RT‐PCR‐negative and non‐COVID‐19 patients. Other results approaching significance include ALP and eGFR.

**Discussion:**

Patients suffering from COVID‐19 and diabetes concurrently can suffer from profound metabolic disturbance, with a significant difference in inpatient mortality. However further, prospective detailed investigation into biochemical processes is needed to fully elucidate underlying mechanisms that affect these patients' outcomes.

## Graphical abstract

This is the first study to investigate diabetic emergencies in the inpatient setting during the first wave of the COVID‐19 pandemic. We demonstrate a mortality difference in patients with diabetes, and highlight the importance of careful observation of patients suffering from both COVID‐19 and a diabetic emergency.

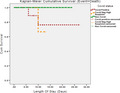

## 1 INTRODUCTION

Severe Acute Respiratory Syndrome Coronavirus 2 (SARS‐COV‐2) is a novel coronavirus that has triggered a global pandemic of which the current trajectory remains uncertain. Early data suggest that increasing age and cardiovascular disease are the markers for severity and mortality of the Coronavirus Disease 2019 (COVID‐19).^1^ A third of deaths in the United Kingdom with COVID‐19 occurred in people with diabetes: 31.4% in people with type 2 diabetes, 1.5% in those with type 1 diabetes and 0.3% in people with other types of diabetes.^2^ Recent epidemiological studies have suggested that risk of mortality in COVID‐19 can be up to 50% higher in patients with diabetes,^2^ and a recent review has suggested that obese patients are at high risk of mortality from COVID‐19. Hyperglycaemia and a known history of diabetes were independent predictors of death in a large cohort of patients with SARS‐COV,^3^ further suggesting that diabetes could be of significance in the disease course of COVID‐19. A recent meta‐analysis also showed that diabetes is associated with mortality, severe COVID‐19 acute respiratory distress syndrome (ARDS) and disease progression.^4^

Bornstein et al. recently published a consensus statement on the management of diabetes with COVID‐19 and suggested that there could be an increase in Diabetic Ketoacidosis (DKA) in type 1 diabetes (T1DM) and type 2 diabetes (T2DM) on sodium glucose cotransporter inhibitors (SGLT2). They also report that there is an increased frequency DKA presentations, which might partly be due to delayed presentation.^5^ Diabetes UK have also recently published guidelines on the management of both hyperglycaemia and DKA that include advice to stop metformin and SGLT2 inhibitors for all suspected COVID‐19 with diabetes.^6^

Diabetic emergencies most associated with infection are DKA and Hyperglycaemic Hyperosmolar State (HHS).^5^, ^7^ A recent study from China has shown that COVID‐19 does cause ketosis and ketoacidosis requiring prolonged hospital admission. Interestingly ketosis was present in both patients with and without diabetes, and the reported data does not distinguish between Type 1 and Type 2 diabetes.^11^ The CORONADO study, investigating the phenotypic characteristics and prognosis of diabetes patients with COVID‐19, suggested that younger, type 1 diabetes patients have a lower risk of severe COVID‐19 prognosis.^8^

There is little evidence available regarding the management of patients with diabetes who are also suffering from COVID‐19. Uncontrolled hyperglycaemia and diabetes, of either type, were recognized as significant risk factors for both severity and mortality for different viruses, including other novel coronaviruses akin to COVID‐19.^9^

Our aim was to report on the incidence, clinical course and outcomes from our cohort of patients who are being hospitalized for COVID‐19 and a diabetic emergency.

## 2 METHODS

We conducted a retrospective case analysis of patients with diabetes admitted to the medical wards of North Middlesex Hospital, London, during the COVID‐19 pandemic between 15 March and 15 April 2020. The inclusion criteria were: (1) positive swab testing for COVID‐19, (2) admission due to a diabetic emergency. We recorded details regarding the length of hospital stay, features of the metabolic emergency, time until resolution, treatment pathways utilized, clinical course of the patient, clinical outcome at 7 days post admission. Data for the study were extracted from routine hospital records. Notes were manually reviewed for results as well as for follow‐up information. The study was part of a departmental audit, evaluating treatment pathways for patients with diabetic emergencies during the pandemic, and has been approved by the Ethics Department of our Hospital.

### 2.1 Diagnosis of COVID‐19

Primarily patients with clinical features matching the UK government case definition for COVID‐19,^10^ or patients who had biochemical/radiological features of COVID‐19 received one COVID‐19 PCR swab at the time of admission. During the time of this study, our local policy was not to re‐swab patients if the PCR swab result was negative, despite a high clinical suspicion. Clinical suspicion was based on symptomatic, biochemical or radiological evidence consistent with COVID‐19.

### 2.2 Diagnostic criteria for diabetic emergencies

DKA: all of^1^ blood glucose concentration >11mmol/l or known to have diabetes,^2^ blood ketones >3.0 mmol/l,^3^ venous pH <7.3 and/or bicarbonate concentration <15mmol/l^11^

HHS: clinically hypovolaemic with markedly raised blood glucose concentration (>30 mmol/l), without hyperketonaemia (ketones <3 mmol/l) or acidosis (pH >7.3) with raised osmolality (> 320 mosmol/kg)^12^

Mixed DKA/HHS: clinical overlap state where features are present from the above clinical syndromes.

### 2.3 Control group

We also had a group of patients who underwent treatment for a diabetic emergency without being investigated or treated for COVID‐19. These patients were used as comparators to investigate significant metabolic differences.

### 2.4 Statistical analysis

Data were stored on a custom, anonymized Microsoft Excel 2010 (Microsoft, Richmond VA) database. Statistical analysis was done using Statistical Package for Social Sciences version 25.0 (SPSS). Qualitative characteristics were presented as mean values ±standard deviation while quantitative characteristics were presented as percentages (%). Differences between groups were evaluated using independent student t‐test. Statistical significance was set at the level of p‐value <0.05. We have split the patients into RT‐PCR swab positive and RT‐PCR swab negative but treated with a high clinical suspicion. Kaplan–Meier survival graph was calculated based on length of stay in days for the three patient groups with death as the event.

## 3 RESULTS

Thirty patients are included in this retrospective analysis, with their demographics demonstrated in Table 1. Twenty‐one (70%) of these patients received treatment for both COVID‐19 and a diabetic emergency during their admission. Two patients with COVID‐19 needed an ICU (intensive care unit) admission, one of whom died. This mortality was also COVID‐19 RT‐PCR swab positive.

**TABLE 1:** Baseline Demographics of all patients that were management for both a diabetic emergency and COVID‐19, including destination at 7 days

	COVID−19 Swab Positive (*n* = 14)	COVID−19 Swab Negative, High Clinical Suspicion of COVID−19 (*n* = 7)	Non‐COVID−19 patients (*n* = 9)
Age (years), mean ± SD	51.21 ± 17.92	47.33 ± 20.46	42.00 ± 18.83
Gender, *n* (%)			
Male	12 (85.7)	5 (71.4)	4 (44.4)
Female	2 (14.3)	2 (28.6)	5 (55.6)
Ethnicity, *n* (%)			
African	4 (28.6)	4 (57.1)	2 (22.2)
Asian	4 (28.6)	1 (14.2)	0 (0.0)
White British	1 (7.1)	1 (14.2)	4 (44.4)
Caucasian	3 (21.4)	1 (14.2)	3 (33.3)
Mixed	1 (7.1)	0 (0.0)	0 (0.0)
Unknown	1 (7.1)	0 (0.0)	0 (0.0)
Diabetes Diagnosis, *n* (%)			
Type 1	1 (7.1)	3 (42.9)	5 (55.6)
Type 2	12 (78.6)	4 (57.1)	4 (44.4)
New Diagnosis	1 (7.1)	0 (0.0)	
Diabetes Emergency, n (%)			
DKA	5 (35.7)	1 (14.2)	9 (100.0)
HHS	1 (7.1)	2 (28.6)	0 (0.0)
Mixed DKA/HHS	3 (21.4)	1 (14.2)	0 (0.0)
Hyperglycaemic Ketosis	5 (35.7)	1 (14.2)	0 (0.0)
Redeveloped Emergency	0 (0.0)	0 (0.0)	0 (0.0)
Pre‐Admission Medication, *n* (%)			
Metformin	8 (57.1)	5 (71.4)	3 (33.3)
DDP4 inhibitors	1 (7.1)	1 (14.2)	1 (11.1)
Insulin	7 (50.0)	3 (42.9)	6 (66.7)
Other hypoglycaemics	4 (28.6)	2 (28.6)	1 (11.1)
No prior medication/Diet controlled	4 (28.6)	1 (14.2)	0 (0.0)
ICU Admissions, n (%)	2 (14.3)	1 (14.2)	2 (22.2)
Destination at 7 days, n (%)			
Discharged	5 (35.7)	6 (85.7)	9 (100.0)
Ward	7 (50.0)	0 (0.0)	0 (0.0)
ICU	1 (7.1)	0 (0.0)	0 (0.0)
Died	1 (7.1)	1 (14.2)	0 (0.0)

Abbreviations: DKA, diabetic ketoacidosis; HHS, Hyperosmolar Hyperglycaemic State; Other oral hypoglycaemics include sulfonylureas, glucagon‐like peptide 1 agonists; ICU, Intensive Care Unit.

Fourteen of 30 included patients were RT‐PCR‐positive patients. 79% (*n* = 2) had type 2 diabetes. 86% (*n* = 12) of RT‐PCR‐positive patients were males and 14% (*n* = 2) females with average age of 51.47 ± 17.30 years. Five patients presented with DKA, one with HHS, three with a mixed DKA/HHS picture and five with hyperglycaemic ketosis. In terms of ethnicity, there were patients who were African (*n* = 5), White British (*n* = 1), Caucasian and Asian (including Eastern and Southern Asia) (*n* = 6). 47% of RT‐PCR‐positive patients were discharged home within 7 days.

Seven patients were RT‐PCR negative but treated for COVID‐19 due to high degree of clinical suspicion. The average age was 47.33 ± 20.46 years, with five males and two females. 43% of these patients had type 1 diabetes. One patient had DKA, two had HHS, two had a mixed picture with one patient having hyperglycaemic ketosis. 67% of patients had been successfully discharged home within 7 days.

For the patients who were not treated for COVID‐19 (*n* = 9), all patients had DKA despite the fact that 33% had type 2 diabetes. There were four males and five females. 67% (n=6) of these patients were insulin treated, and 100% were discharged home within 7 days of admission.

Table 2 demonstrates the admission gas results, blood tests and glycosylated haemoglobin (HbA1c) as well as selected COVID‐19 markers. Broadly routine admission blood tests were similar when averaged and compared between the three groups. There was a non‐significant rise in white cell count going from the RT‐PCR patients to non‐COVID‐19 patients. No significant lymphopaenia was observed between any of the three groups.

**TABLE 2:** Admission Blood test results including some COVID‐19 markers

Blood test result mean ± SD	COVID−19 Swab Positive (*n* = 14)	COVID−19 Swab Negative, High Clinical Suspicion of COVID−19 (*n* = 7)	COVID−19 Swab Negative, Low Clinical Suspicion of COVID−19 (*n* = 9)	*p^a^*	*p^b^*	*p^c^*
Admission blood glucose (mmol/l),	23.86 ± 8.38	33.29 ± 11.49	26.34 ± 12.47	.**045**	.573	.272
Serum ketones (mmol/l)	4.44 ± 2.53	5.6 ± 2.51	5.67 ± 0.99	.314	.154	.943
Time for ketone resolution (h)	43.10 ± 44.01	25.70 ±15.81	28.44 ± 20.60	.413	.378	.794
Admission HbA1c (mmol/mol)	115.30 ± 42.85	91.83 ± 12.27	109.75 ± 46.95	.217	.834	.387
Pre‐admission HbA1c (mmol/mol)	99.38 ± 31.18	70.60 ± 39.76	73.33 ± 15.70	.131	.210	.893
Haemoglobin (g/l)	139.92 ± 24.63	143.86 ± 18.45	134.67 ± 39.42	.717	.703	.580
WCC (×10^9^/l)	10.06 ± 3.46	10.37 ± 4.16	12.54 ± 4.31	.857	.149	.328
Platelets (×10^9^/l)	281.08 ± 94.39	283.71 ± 76.84	296.44 ± 103.53	.950	.722	.790
Lymphocytes (x10^9^/l)	1.25 ± 0.55	1.32 ± 0.77	1.26 ± 0.95	.814	.953	.909
Sodium (mmol/l)	141.08 ± 13.08	155.43 ± 38.33	134.11 ± 9.07	.229	.183	.126
Potassium (mmol/l)	5.28 ± 0.82	5.51 ± 1.13	5.22 ± 1.35	.601	.913	.653
Urea (mmol/l)	13.41 ± 12.73	15.47 ± 6.74	15.16 ± 13.16	.696	.758	.955
Creatinine (micromoles/litre)	183.77 ± 186.70	179.71 ± 48.96	122.67 ± 52.29	.956	.354	.**043**
eGFR	53.67 ± 30.09	41.86 ± 16.38	73.22 ± 27.25	.224	.580	.089
CRP (mg/l)	88.32 ± 68.07	26.83 ± 24.35	57.40 ± 123.91	.**026**	.459	.485
Ferritin (ng/ml)	1321 ± 1015.79	921.67 ± 432.28	–			
LDH (U/L)	391.60 ± 227.32	414.33 ± 164.69	–			
Bilirubin (micromole/litre)	5.85 ± 3.34	6.43 ± 3.60	7.00 ± 4.64	.721	.504	.792
ALT (iu/l)	34.00 ± 49.96	28.85 ± 11.87	25.50 ± 16.03	.452	.244	.657
ALP (iu/L)	92.23 ± 32.24	86.14 ± 46.52	125.67 ± 60.13	.734	.106	.174
CK (U/L)	2033.50 ± 2035.76					
INR	1.87 ± 2.28	1.10 ± 0.09	1.07 ± 0.07	.434	.411	.415
PT	17.87 ± 19.24	11.48 ± 0.92	11.27 ± 0.68	.437	.422	.653
APTT	37.00 ± 24.89	27.833 ± 4.26	31.00 ± 3.29	.395	.572	.180
Blood gas						
pH	7.24 ± 0.17	7.18 ± 0.22	7.06 ± 0.18	.533	.**028**	.263
HCO3−	15.79 ± 7.40	13.33 ± 8.73	9.23 ± 6.36	.506	.**040**	.295
Lactate	3.70 ± 6.18	3.08 ± 0.40	3.22 ± 2.38	.813	.828	.891

The values in bold were found to be statistically significant. Abbreviations: ALP, Alkaline Phosphatase; ALT, Alanine Transaminase; APTT, activated partial thromboplastin time; CK, Creatine Kinase; CRP, C‐reactive protein; eGFR, estimated glomerular filtration rate; INR, International Normalized Ratio; PT, Prothrombin Time; WCC, white cell count.

p^a^—difference between COVID‐19 RT‐PCR‐positive and COVID‐19 RT‐PCR‐negative patients; p^b^—difference between COVID‐19 RT‐PCR‐positive and non‐COVID‐19 patients; p^c^—difference between COVID‐19 RT‐PCR‐negative patients and non‐COVID‐19 patients.

Admission pH was not significantly different between RT‐PCR positive and negative patients (*p *= .533); however, there was a significant difference between RT‐PCR‐positive COVID‐19 patients and our non‐COVID‐19 patients (*p *= .028). There was a significant difference between RT‐PCR‐positive COVID‐19 patients and non‐COVID‐19 patients with regard to HCO3 (*p *= .04). There was a significant difference in the C‐reactive protein (*p*=0.026) and glucose (*p *= .045) between RT‐PCR positive and negative patients.

**FIGURE 1:**
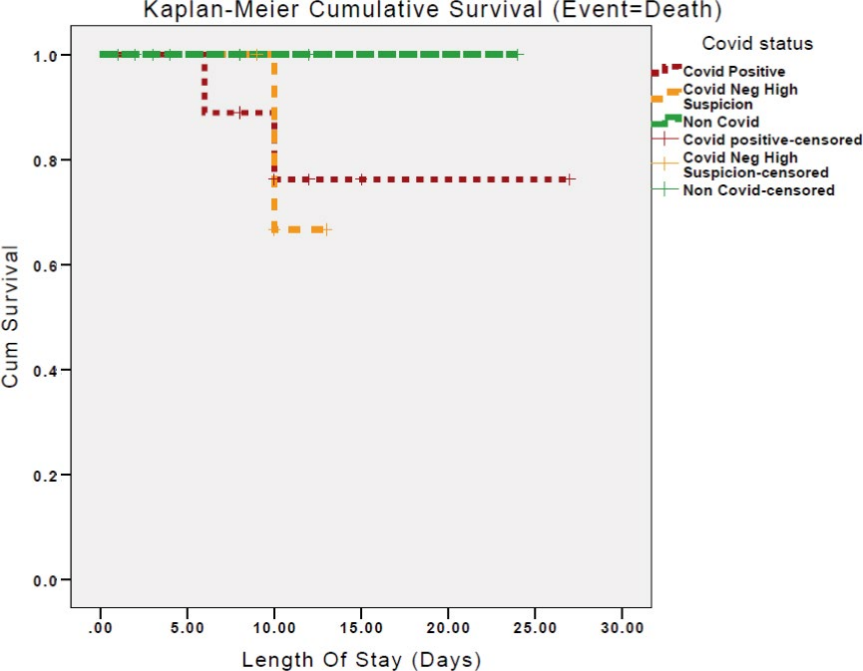
Kaplan–Meier survival curves between RT‐PCR‐positive, RT‐PCR‐negative and non‐COVID‐19 patients during their admission

Kaplan–Meier survival curves were drawn for the groups, comparing all‐cause mortality. This is shown in Figure 1. It demonstrates that the cumulative survival time drops if being treated for COVID‐19, and more so if treated for COVID yet RT‐PCR negative.

## 4 DISCUSSION

This is the first single‐centre report on COVID‐19‐related diabetic emergencies. Some of the data in this study were part of the three‐centre study which was published in Lancet Diabetes and Endocrinology recently.^13^ We have observed that these patients are profoundly unwell from their diabetic emergencies, with COVID‐19 swab‐negative patients seemingly having greater metabolic disturbance. This could be related to the levels of viraemia; however, this analysis did not have access to any investigations that can accurately quantify this. During their admission, all patients received fluid and insulin therapy as per the DKA or HHS protocols for the trust, with no deviation in patients who also received treatment for COVID‐19.

Most of the results did not achieve statistical significance due to the relatively low number of subjects in each arm. Of interest in this study is that the high percentage of people of African and Asian ethnicity who presented with diabetic emergencies, especially ketosis/ketoacidosis. The other interesting observation is the length of time required for ketone resolution was 43 hours for COVID‐19 positive cases as opposed to 23–27 h for COVID‐19‐negative patients, even though this was statistically not significant.

The Kaplan–Meier curve (Figure 1) demonstrated a clear difference in mortality between COVID‐19 patients and the non‐COVID‐19 cohort. Interestingly, the RT‐PCR‐positive group had the worst outcomes which correlates to the greater metabolic dysfunction observed. Although given that these Kaplan–Meier curves were only drawn for 7 days following diagnosis as patients were lost to follow up, it is difficult to ascertain any further effect on the patients. This perceived difference between RT‐PCR‐positive and ‐negative patients may also be due to the efficacy of the swab result itself as some were treated on clinical grounds for COVID‐19. Larger studies would be able to address this issue.

The very high admission HbA1c in all groups suggest poor control in all arms. This points to the importance of good glycaemic control to prevent mortality and morbidity. North Middlesex Hospital serves two of the very deprived boroughs of London and the high HbA1c observed in this study reflects the need for better detection, education, awareness and the need for more resource allocation for effective glycaemic control in the population served.

In our cohort, we had one patient newly diagnosed with diabetes. Newly diagnosed diabetes with COVID‐19 infection is a topic of increasing interest. A recent review by Sathish et al. demonstrated a pooled proportion of 14.4% in a multinational cohort of 3700 patients.^14^ This could further deepen the impact of COVID‐19 on public health infrastructure as diabetes already has a significant economic burden.^14^

Another interesting observation is the high serum creatinine and corresponding low eGFR in clinically COVID‐19 subjects (PCR positive and negative) as opposed to COVID‐19‐negative patients. This correlates with the observation that acute kidney injury (AKI) is more often observed in this cohort.^15^ There was no difference in liver function tests unlike previous published trials.^16^ C‐reactive protein (CRP) was elevated in RT‐PCR‐positive patients as opposed to the other groups, though again not statistically significant.

Our choice of COVID‐19 markers included ferritin, lactate dehydrogenase, CRP and lymphocytes as indicators of COVID‐19 disease severity. This was based on a retrospective study from Wuhan, the region of China first affected by COVID‐19.^17^ Our patients show elevated levels of these markers, with marginally reduced lymphocyte count, suggesting that there is severe metabolic derangement—however, whether this is due to COVID‐19, the diabetic emergency or a combination is yet to be determined. Similarly, the COVID‐19 markers used at our centre are non‐specific and can be elevated purely from a diabetic emergency point of view. Unfortunately, during this period, the investigation of possible COVID‐19 with certain biomarkers was not within an established protocol meaning that some patients did not have all investigations performed.

### 4.1 Limitations

Our study was small (*n* = 30) which makes statistical analyses difficult and can lead to false significance. However, the findings of our retrospective analysis are corroborated by both Bornstein et al.^5^ and the Diabetes UK guidelines^6^ whose recommendations are in line with our findings.

One other key factor of our analysis revolves around the COVID‐19 RT‐PCR swab status. Twenty‐one patients were treated for COVID‐19 and a diabetic emergency during their admission regardless of swab status. Issues regarding the reliability of the COVID‐19 swabs are known, with high proportion of false negatives and need for interpreting results with caution.^18^, ^19^ However, the only way to date to successfully test for COVID‐19 is by RT‐PCR of nasal swab, tracheal aspirate or bronchoalveolar lavage samples.^20^ There are many barriers to the rapid roll out of serological tests including assessment of sensitivity and specificity of the tests, possibility of false‐positive tests due to cross‐reactivity with other viral pathogens and ensuring that mass production of the test is economically viable.^21^ Another key limitation of this report is that this is a retrospective analysis of routinely collected data, meaning that there are some gaps in investigations. These primarily included lactate dehydrogenase, ferritin and aspartate transaminase in the non‐COVID‐19 patients and some of the patients treated during the COVID‐19 pandemic.

### 4.2 Conclusion

We present a single‐centre observational analysis of COVID‐19 patients who suffered from a diabetic emergency, in comparison to non‐COVID‐19 patients. Some significant differences were found; however, further investigations are needed into the relationship of COVID‐19 and their impact on diabetic control and the risk of diabetic emergencies. A significant difference in mortality was observed. Prospectively collected data with a larger patient base, including data on COVID‐19 markers, could help shed further light on the management of inpatient diabetics with COVID‐19.

## CONFLICT OF INTEREST

Nothing to declare by any author.

## AUTHOR CONTRIBUTIONS

**Tobin Joseph:** Conceptualization (lead); Data curation (lead); Formal analysis (lead); Investigation (lead); Methodology (lead); Validation (lead); Writing‐original draft (lead); Writing‐review & editing (lead). **Eleanor Crawley:** Data curation (supporting); Investigation (supporting); Project administration (supporting). **Sulmaaz Qamar:** Data curation (supporting); Methodology (supporting); Project administration (supporting); Supervision (supporting); Writing‐original draft (supporting). **Maya Zosmer:** Data curation (supporting); Investigation (supporting); Project administration (supporting); Validation (supporting). **Girish Rayanagoudar:** Conceptualization (supporting); Supervision (supporting); Writing‐original draft (supporting). **Chirdambaram Nethaji:** Conceptualization (supporting); Supervision (supporting); Writing‐original draft (supporting). **Ravi Menon:** Conceptualization (lead); Formal analysis (lead); Supervision (lead); Visualization (lead); Writing‐original draft (lead); Writing‐review & editing (lead).

## ETHICS APPROVAL AND CONSENT TO PARTICIPATE

We followed the NHS REC decision flowchart and in line with this guidance ethical approval was not sought. The NHS REC flowchart can be found at http://www.hra‐decisiontools.org.uk/ethics/. We felt that this vital information should be shared with the wider medical community and so a manuscript was prepared and submitted to BMC. Consent was not sought at the time of patient admission as they were not being enrolled into any study. This was part of a clinical audit registered with the trust for review of paper notes. All patient healthcare data were collected anonymously, retrospectively and no patient identifiable data were collected or described in the manuscript. This was in line with the NHS Health Research Authority guidance.^22^

## Data Availability

Available from authors on reasonable request.
